# Scythes, sickles and other blades: defining the diversity of pectoral fin morphotypes in Pachycormiformes

**DOI:** 10.7717/peerj.7675

**Published:** 2019-11-07

**Authors:** Jeff J. Liston, Anthony E. Maltese, Paul H. Lambers, Dominique Delsate, William E.H. Harcourt-Smith, Anneke H. van Heteren

**Affiliations:** 1 Vertebrate Palaeontology, SNSB-Bayerische Staatssammlung für Paläontologie und Geologie, Munich, Bavaria, Germany; 2 Palaeobiology, Department of Natural Sciences, National Museums Scotland, Edinburgh, Scotland; 3 School of Earth Sciences, University of Bristol, Bristol, England; 4 Institute of Biodiversity, Animal Health and Comparative Medicine, College of Medical, Veterinary and Life Sciences, University of Glasgow, Glasgow, Scotland; 5 Rocky Mountain Dinosaur Resource Center, Woodland Park, CO, USA; 6 Universiteitsmuseum Utrecht, Utrecht University, Utrecht, Netherlands; 7 Centre de Recherche Scientifique/Paléontologie, Musée National d’histoire Naturelle de Luxembourg, Luxembourg, Luxembourg; 8 Division of Paleontology, American Museum of Natural History, New York, NY, USA; 9 Department of Anthropology, Lehman College, City University of New York, New York, NY, USA; 10 Department of Anthropology, The Graduate Center, City University of New York, New York, NY, USA; 11 Sektion Mammalogie, Zoologische Staatssammlung München, Staatliche Naturwissenschaftliche Sammlungen Bayerns, Munich, Germany; 12 GeoBio-Center, Ludwig-Maximilians-Universität München, Munich, Germany; 13 Department Biologie II, Ludwig-Maximilians-Universität München, Munich, Germany

**Keywords:** Pachycormidae, Aspect ratio, Character creep, Planform, Leedsichthys, Bonnerichthys, Protosphyraena, Pectoral fin morphotype, Saurostomus, Lifestyle indicator

## Abstract

The traditional terminology of ‘scythe’ or ‘sickle’ shaped is observed to be flawed as an effective descriptor for pectoral fin shape in pachycormids. The diversity of pachycormid pectoral fin shapes is assessed across the 14 recognised genera that preserve complete pectoral fins, and improved terms are defined to more effectively describe their form, supported by anatomical observation and aspect ratio analysis of individual fins, and corroborated by landmark analysis. Three clear and distinct pectoral fin structural morphotypes emerge (falceform, gladiform, falcataform), reflecting a diversity of pachycormid lifestyles throughout the Mesozoic, from agile pursuit predator to slow-cruising suspension feeder.

## Introduction

### Pachycormid problems

Pachycormids occupy a key-position within Actinopterygii, as part of the Holostei-Teleostei transition. Their precise position in this hierarchy has been disputed for several years, ever since Patterson’s work (1973). Furthermore, the group remains highly problematic due to a large number of historical problems with both descriptions (poorly defined genera and species) and material (holotypes based on incomplete or missing specimens), which undermines confidence in recent phylogenetic analyses (Ferrón et al., 2018), notwithstanding the high percentage of characters coded as unknown in such datasets (Arratia & Schultze, 2013) even greater than those quoted for the genus *Pachycormus* (López-Arbarello & Sferco, 2018). The issue relating to material is deeply affected by the trend within the group for reduced skeletal ossification, particularly concerning their body scales and vertebrae (arcocentra and chordacentra), which can severely compromise the cohesiveness and thus the collectability of some Jurassic and many Cretaceous pachycormid taxa (Gouiric-Cavalli et al., 2019).

A number of the earliest described taxa within Pachycormiformes were described on the basis of poor material (Quenstedt, 1858; Agassiz, 1833), despite the fact that the genera are best known from the Holzmaden and Solnhofen lithologies, both with global reputations for fine preservation. For example, *Pachycormus bollensis* is defined on the basis of a single dentary, that can no longer be found in the collections at Tübingen (L. Wretman, 2017, personal communications). Clarity over the definitions of pachycormid taxa (as with many other fossil taxa) has not been improved by the number of type specimens destroyed through bombing during wartime (Liston & Gendry, 2015), which has introduced a need for neotype material to be identified (e.g. *Asthenocormus titanius*, *Hypsocormus macrodon*, *Protosphyraena tenuis*). Lambers (1992), as well as highlighting the need for neotype designations for some pachycormid taxa, similarly noted problems of original type material quality with both *Pachycormus* and *Hypsocormus*.

The pervasive reduced ossification throughout the clade also compromises the chances of material being preserved. Indeed, it is noteworthy that in spite of its global reputation for preserving feathered animals like *Archaeopteryx*, the Solnhofen lithographic limestone seems generally poor in terms of the preservation of pachycormid fish material, in sharp contrast to the Holzmaden Posidonienschiefer, which may reflect different problems of matrix preparation for fish with restricted skeletal ossification. The largest adult-sized pachycormiform taxa are the least complete, as demonstrated by some 70 fragmentary skeletons of the suspension-feeding *Leedsichthys* (Liston, 2010), and the fact that it took almost 140 years after the genus was first described, before a single articulated specimen of *Protosphyraena* was found (included in this study). The reduced ossification is a particular problem, as Arthur Smith Woodward noted the small scale size (the thin rhombic scales noted as defining the ‘microlepidoti’ of Wagner, 1851; Heineke, 1906) and unusually high segmentation in the notochord as defining features in many genera of the Family Pachycormidae (1895), and clearly the preservability of both of these features is significantly compromised by limited ossification. Intriguingly, in this regard, the reduced ossification of vertebral components reveals an unconventional cranial direction of ossification during development, which apparently has only been observed otherwise in the early tetrapod *Whatcheeria* (J. Clack, 2012, personal communication).

As a result of this reduced axial ossification, the earliest pachycormid remains noted in 18th century collections (Liston, 2015) were isolated pectoral fins. Liston (2008) first drew attention to the fact that the long referred to ‘scythe’-shaped pectoral fin was not in fact a pachycormid synapomorphy, with particular reference to *Asthenocormus*, going on to identify a problem of tacitly assumed rather than observed character repetition across a group (Liston et al., 2012), later echoed by Arratia & Schultze (2013). Indeed, when the Family Pachycormidae was established by Woodward (1895), his terminology was different, describing the pectoral fin as ‘sickle’ shaped, and explicitly stating this only for the genera *Asthenocormus*, *Pachycormus*, *Hypsocormus* and *Protosphyraena*. (*Sauropsis*, *Prosauropsis*, *Euthynotus* and provisionally *Leedsichthys* were also noted as included within the Family, but with no such statement about their pectoral fins.) In tracing the development of this ‘meme’ within the scientific literature, it can be seen that Patterson appears to have introduced the error: ‘The pachycormids are undoubtedly a monophyletic group, characterised by…large, scythe-like pectoral fins’ (1973: p.273). Through an apparent ‘slip of the pen’, Woodward’s original description transformed from one bladed agricultural implement to a very different one, apparently going unnoticed until Liston (2008). Subsequently, Mainwaring (1978; supervised by Patterson) perpetuated this mistake within the first substantive cladistic analysis of the pachycormids (*Prosauropsis* and *Leedsichthys* were removed by Mainwaring from the group in her study), in which she stated that: ‘The scythe-like shape of the pectoral fins (character 5) has been recognised as a pachycormid specialisation almost since the creation of the family’ (1978: p.114). Mainwaring’s thesis work and character phrasing were later entrenched in the literature via Lambers’ subsequent revision of Mainwaring’s work as part of his review of Solnhofen fishes (1992). From there, subsequent authors appear to have faithfully recorded the presence of a ‘scythe-like’ pectoral fin in any pachycormids that they described (in spite of the recognition of *Neopachycormus* as an unrecognised plethodid tselfatiiformid (Taverne & Liston, 2017), the number of pachycormid genera has doubled since Lambers’ review), without necessarily giving any serious consideration to what the term meant (Friedman, 2012). This has resulted in a phenomenon of ‘character creep’, where the integrity of the original definition deforms through casual author use over time (in a similar way to ‘name drift’ as with e.g. the spelling of *Cryptoclidus*, Brown, 1981), until it becomes virtually meaningless, a problem that occurs in many other datasets such as those of basal sauropodomorphs (O. Regalado Fernandez, 2018, personal communication). Perhaps ironically, a recent paper has actually reverted to using Woodward’s original descriptor of ‘sickle’-shaped (Přikryl et al., 2012) for pachycormid specimens.

This work aims to demonstrate the wide diversity of pectoral fin shape amongst pachycormid taxa, reflecting a range of ecological spaces occupied by the members of this family, through comparison of planform and aspect ratio (AR), and finally define useful categories for describing and defining this variability in pectoral fin shape.

## Materials and Methods

**Figure 1:**
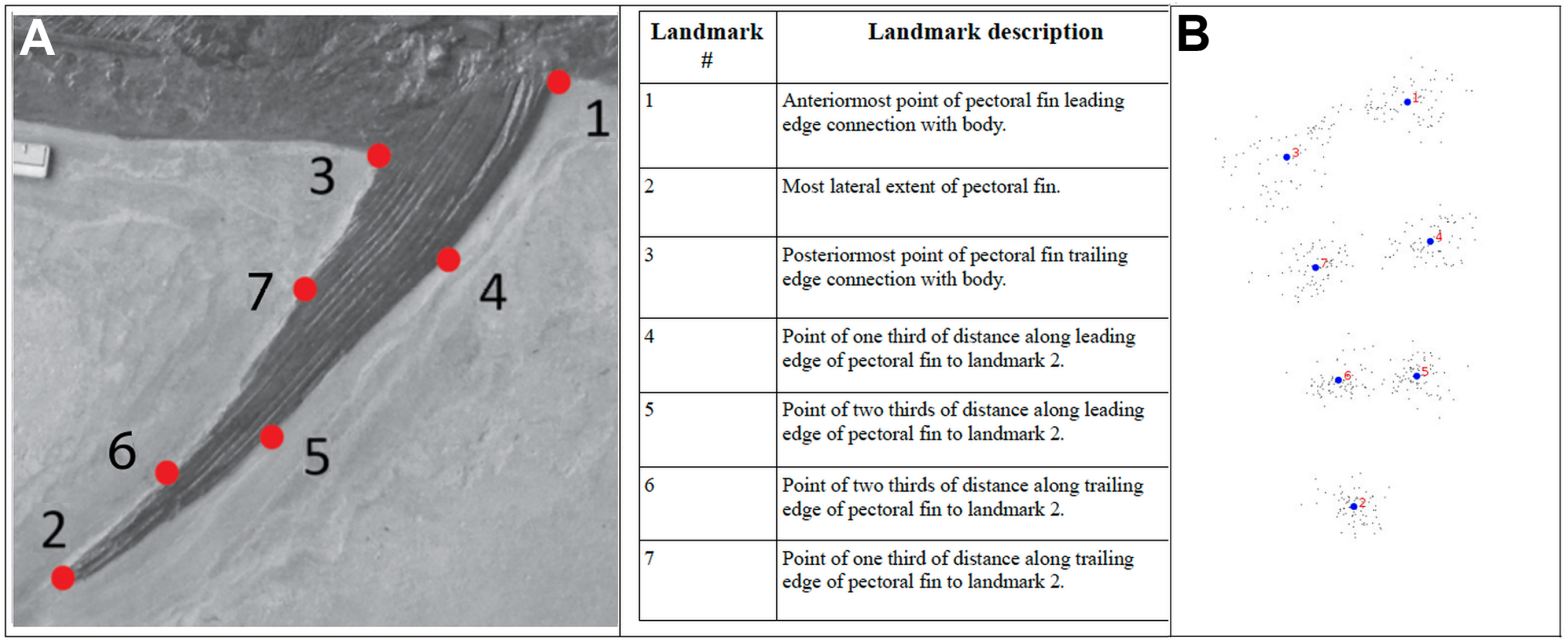
Description of Landmarks. Relative positions of landmarks shown (A) on photograph of example specimen and (B) as a pointcloud for all specimens. In both cases, the leading edge of the pectoral fin is at the right, with the trailing edge of the pectoral fin at the left. The photograph of the left pectoral fin of IRSNB Vert-00-133 (see Fig. 8) has been reversed for consistency with the pointcloud.

This study was initiated to assess the degree of variability in pectoral fin planform across (and within) pachycormid taxa, using hydrodynamically relevant parameters. It was anticipated that pectoral fins would have forms with hydrodynamic properties reflecting the niches and lifestyles of the individual taxa. Pectoral fin specimens were photographed with a scale in standard orientation, with the camera pointing perpendicular to the anterior–posterior plane. Photographs of all specimens were then examined in ImageJ in terms of their length, surface planform area and AR. This was derived from the span of the fin squared, divided by the fin surface area (following the methodology in Lighthill, 1977), the pectoral fin surface area and the span of each fin being measured in ImageJ, the span being taken from the midpoint of the pectoral fin/body junction (as indicated by the origins of the pectoral lepidotrichia) to the tip of the pectoral fin. These data were then processed in Microsoft Excel and OpenOffice Calc to calculate the AR, and the results tabulated in Table 1. An initial assessment was then carried out to see if the observed diversity grouped into morphotypes. These morphotypes were then assessed using 2D geometric morphometrics, with homologous *x*, *y* landmark coordinates (see Fig. 1 for visual and anatomical descriptions of the landmarks) and scale data being collected from the digital images using the software tpsDig2w32 (http://life.bio.sunysb.edu/morph/), and exported into text file format. Coordinate data were then subjected to a generalized procrustes analysis (GPA) in the software MorphoJ (Klingenberg, 2011). GPA is a superimposition technique that corrects for translational and rotational differences and adjusts for size. GPA aligned data were analysed using principal components analysis (PCA) and discriminant function analysis (DFA), also using MorphoJ. Regression analyses were performed in the software package PAST 3.2 (Hammer, Harper & Ryan, 2001).

**Table 1:** Results of pectoral fin survey in selected pachycormids. Specimen number along with genus, aspect ratio and pectoral fin morphotype.

Genus	Specimen/collection number	A/R	Fin type
*Haasichthys*	Tu228 (holotype)	3.06	1
*Orthocormus*	T.14836 (holotype)	4.17	1
*Orthocormus*	JM-E SoS Scha 2418	4.66	1
*Orthocormus*	SenkM 1863 (holotype)	1.53	1
*Orthocormus*	BSPG.1993.XVIII-VFKO.B16 (holotype)	2.67	1
*Saurostomus*	NHMUK OR3731 L	5.6	1
*Saurostomus*	SMNS 51144	2.96	1
*Saurostomus*	SMNS 56982	3.48	1
*Saurostomus*	SMNS 50736 R	2.04	1
*Saurostomus*	SMNS 50736 L	2.86	1
*Saurostomus*	NHMUK PV P.11126	4.52	2
*Pachycormus*	SMNS 51041	2.1	1
*Pachycormus*	SMNS 55300	4.02	1
*Pachycormus*	SMNS 51905 L/R	6.28/3.14	1
*Pachycormus*	SMNS 51199 L	4.46	1
*Pachycormus*	SMNS 51199 R	4.1	1
*Pachycormus*	SMNS 56230	2.94	1
*Pachycormus*	SMNK-PAL.6680	3.77	1
*Pachycormus*	PA-49.1911	3.03	1
*Pachycormus*	SMNS 54835	3.59	1
*Pachycormus*	NHMUK PV P.12913	1.34	1
*Pachycormus*	PMU24798	4.68	1
*Pachycormus*	GLAHM V7274	4.38	1
*Pachycormus*	PMU24796	4.92	1
*Pachycormus*	SMNS 55857 R	4.66	1
*Pachycormus*	SMNS 55857 L	5.3	1
*Pachycormus*	SMNS 6696 L/R	6.35/4.24	1
*Pachycormus*	SMNS 87762 R	4.22	1
*Pachycormus*	SMNS 15815 L	2.33	1
*Pachycormus*	SMNS 15815 R	2.03	1
*Pachycormus*	IRSNB Vert-00-133	10.05	1
*Pachycormus*	NHMUK PV P.51667	3.54	1
*Pachycormus*	SMNS 54835	3.58	1
*Pachycormus*	NHMUK OR 20657	4.01	1
*Pachycormus*	NHMUK PV P.7569	3.97	1
*Pachycormus*	GPIT/OS/777	3.97	2
*Pachycormus*	SMNS 58389	6.36	2
*Pachycormus*	G.338-1894	3.06	2
*Pachycormus*	SMNS 95430	3.33	2
*Pachycormus*	Ge 30 177	3.86	2
*Pachycormus*	MBF 12215	2.4	2
*Pachycormus*	SMNS 95835 R	3.43	2
*Pachycormus*	SMNS 95835 L	3.48	2
*Sauropsis*	BSP.AS.VII.1089	6.44	2
*Sauropsis*	NHMUK PV P.13007 (holotype)	3.89	2
*Sauropsis*	CM 4772	4.69	2
*Sauropsis*	Tu147	2.32	2
*Euthynotus*	MNHNP 821	3.16	2
*Euthynotus*	MNHNP 10537	3.31	2
*Euthynotus*	MNHNP 10538	3.97	2
*Euthynotus*	AMNH 7540	3.82	2
*Ohmdenia*	GPIT 1017/1 (holotype)	3.84	2
*Leedsichthys*	PETMG F.174	4.2	2
*Martillichthys*	NHMUK PV P.61563 (holotype)	5.9	2
*Hypsocormus*	NHMUK PV P.6011 L	3.88	2
*Hypsocormus*	NHMUK PV P.6011 R	4.03	2
*Hypsocormus*	NMS 1892.55.2	5.42	2
*Hypsocormus*	BSPG.1964.XXIII.524 Schernfeld	2.21	2
*Hypsocormus*	NHMUK PV P.6942 R	4.1	2
*Hypsocormus*	JM-E SoS 539	2.83	2
*Hypsocormus*	JM-E SoS 3916	4.38	2
*Asthenocormus*	Baj2344	2.96	2
*Asthenocormus*	JM-E SoS 542 (neotype)	3.84	2
*Pseudoasthenocormus*	CM 5399	3.86	2
*Pseudoasthenocormus*	BSP.1956.I.361	4.1	2
asthenocormid (TUR)	UANL-FCT 0087	4.74	2
Kimmeridgian juvenile	K.1556	3.99	2
*Bonnerichthys*	UNSM 88507	4.58	2
*Bonnerichthys*	FHSM VP-212	5.51	2
*Bonnerichthys*	FHSM VP-17428 fin 1	6.87	2
*Bonnerichthys*	FHSM VP-17428 fin 2	6.33	2
*Bonnerichthys*	KUVP 465 R	5.24	2
*Bonnerichthys*	KUVP 465 L	5.49	2
*Bonnerichthys*	KUVP 60692	6.19	2
*Bonnerichthys*	RMDRC 14-017	6.87	2
*Protosphyraena*	FHSM VP-80 R	14.83	3
*Protosphyraena*	AMNH FF 21651 L/R	16.10/14.33	3
*Protosphyraena*	RMDRC 11-025	13.12	3
*Protosphyraena*	RMDRC 14-005	20.05	3
*Protosphyraena*	RMDRC 15-020	10.11	3
*Protosphyraena*	RMDRC 03-007	13.72	3
*Protosphyraena*	RMDRC 03-005	11.26	3
*Protosphyraena*	RMDRC 03-006	14.71	3
*Australopachycormus*	NHMUK PV P.73611	8.07	3

As postcranial variability within the genus *Pachycormus* had been identified as unusually large by Liston (2007), the comparatively large number of qualifying representatives of this genus (forming around a third of the specimens) were all accepted for the analysis, in an attempt to bring to light any possible underlying anatomical patterns that might indicate a degree of taxonomic diversity outwith the current recognised species of *bollensis*, *macropterus* and *curtus*, recently critically reassessed by Wretman, Blom & Kear (2016). In relation to this question on a broader scale, *Saurostomus* specimens were also more highly represented, as these two genera have historically been argued to be congeneric (Giebel, 1848). Woodward was undecided on this matter (the type material of *Saurostomus esocinus* being a lost dentary) for the purposes of his catalogue, and acknowledged his uncertainty (1895, 1896a) until finally concluding that they should indeed be separate genera (1896b).

### Specimen selection, including comments on individual taxa

Over 200 pachycormid specimens were assessed from collections around the world, ranging from the Toarcian to the Campanian in age, covering all known genera and utilising type specimens where possible, as well as undescribed material (Blanco-Piñón et al., 2002; Liston, 2012). Eventually, 88 specimens were selected as suitable for analysis (see Table 1 for specimen numbers of selected material of all taxa): to be eligible for this study, specimens had to have at least one pectoral fin, which prevented the inclusion of both *Notodectes argentinus* and *Rhinconichthys* in the analysis (Gouiric-Cavalli & Cione, 2015; Schumacher et al., 2016). From the original pool, 74 specimens (Table 2) were selected for shape analysis, for which at least one well-preserved pectoral fin was required to be present.

**Table 2:** Genus sample sizes for shape analysis.

Taxon	*N*
*Pachycormus*	28
*Saurostomus*	6
*Pseudoasthenocormus*	2
*Hypsocormus*	6
*Sauropsis*	4
*Bonnerichthys*	7
*Leedsichthys*	1
*Martillichthys*	1
*Asthenocormus*	2
*Protosphyraena*	7
*Australopachycormus*	1
Undescribed Kimmeridgian	1
Undescribed Turonian	1
*Haasichthys*	1
*Ohmdenia*	1
*Orthocormus*	2
*Euthynotus*	3

*Asthenocormus*: Six recognised specimens were examined of this genus, with an additional suspected seventh from the Kimmeridgian of Mexico (Liston et al., 2012), but other than the Dresden juvenile specimen, disarticulation of the pectoral fin was almost ubiquitous, rendering analysis problematic relative to the other genera. Foreshortening of the distal ends of the pectoral fins due to either preparation error or preservation flaw was also both common and problematic (JM3556 and L.1309). This meant that only the juvenile and the Neotype adult JM542 qualified for examination, with JM542 less than perfectly clearly defined.

*Australopachycormus*: Given the temporal gap of 10 million years, in addition to the geographical one between Australia and Europe/North America, it was decided to regard this genus as distinct from *Protosphyraena* for the purposes of this study. For this taxon, the most complete pectoral fin is a referred specimen from the NHM collections (NHMUK PV P.73611).

*Bonnerichthys*: The most informative specimen lengthwise is the 4.2 m (from the anterior extent of the skull to the posterior limit of the hypural plate) FHSM 17428 individual. Beyond this, only partial specimens are known of this animal, lacking even paired in situ pectorals, so isolated pectorals were used.

*Euthynotus*: Specimens of both *Euthynotus intermedius* and *E. incognitus* were used.

*Haasichthys*: The holotype specimen (Delsate, 1999) alone was used.

*Hypsocormus*: Lambers (1992) noted many of the problems with this genus, with many of the species not being represented by complete specimens or even complete pectoral fins. The species ‘*macrodon*’ was noted as questionably part of *Hypsocormus*, and it awaits the designation of a Neotype, following the loss of the original designated specimen due to the wartime destruction of collections. The best-preserved specimen of this species, with characteristic asymmetry of the caudal fins, again suffered from pectoral fin foreshortening, so could not be used. Nevertheless, a combination of *Hypsocormus insignis* and *macrodon* specimens were used in this study.

*Leedsichthys*: Although its range has recently been extended to include specimens from Neuquén (Gouiric-Cavalli, 2017), only two specimens have ever been recovered with any pectoral fin sections, but one of those specimens has both fins complete, discovered in an in vivo position. The type specimen does not preserve any pectoral fin material, and was not utilised.

*Martillichthys*: Of the two specimens known, one only preserves stumps of pectoral elements, but the holotype (recently reassessed by Dobson et al., 2019) has one elegantly prepared pectoral fin, the other still lying within the matrix of the collected slabs.

*Ohmdenia*: The holotype is far from complete, but one pectoral fin appeared to be preserved more or less in its original shape, so was able to be used in this analysis, although presenting similar challenges to those noted already for *Asthenocormus*.

*Orthocormus*: The type specimens of the three species (*cornutus*, *teyleri*, *roeperi*) were used, as well as a further Eichstätt museum specimen of *Orthocormus teyleri*.

*Pachycormus*: The type specimen of *Pachycormus bollensis*, an isolated dentary figured by Quenstedt (1858), could not be located within the Tübingen collections. The type specimen of *Pachycormus macropterus* is neither a full-length individual, nor are its pectorals fully preserved to assess surface area and pectoral length. As explained above, a large number of specimens of *Pachycormus* and *Saurostomus* were selected, from collections around the world, but particularly the Stuttgart Museum, with its unrivalled holdings of Holzmaden shale material.

*Protosphyraena*: Only one articulated specimen of *Protosphyraena tenuis* exists, albeit with some rostral damage, that could be used for standard length (SL) based measurements in this study. The length of the missing rostral section was estimated from a similarly-sized *Protosphyraena* skull, LACM 129752. Other isolated pectoral fins of *Protosphyraena perniciosa* and *Protosphyraena nitida* as well as *Protosphyraena tenuis* were, however, used.

*Pseudoasthenocormus*: The nature of the type specimen meant that both pectoral fin foreshortening and uncertainty of SL due to the broken nature of the constituent slabs rendered this specimen ineligible for this study. The Munich specimen, lacking a tail, could also not be used for SL, but was used for AR analysis. A Carnegie specimen (CM 5399) formerly misidentified as *Hypsocormus* ‘*macrodon*’ was reidentified as *Pseudoasthenocormus* during preliminary assessment for this study.

*Sauropsis*: The holotypes of *Sauropsis longimanus* and *Sauropsis veruinalis* were used, but unfortunately the holotype of *Sauropsis depressus* lacked a useable pectoral fin so could not be used.

*Saurostomus*: (see above notes on *Pachycormus*).

During the preliminary assessment of material for this survey, *Neopachycormus birmanicus* was identified as not being a pachycormid, and the taxon was thus recently reassigned to a species of a pre-existing tselfatiid genus (Taverne & Liston, 2017).

## Results

**Figure 2:**
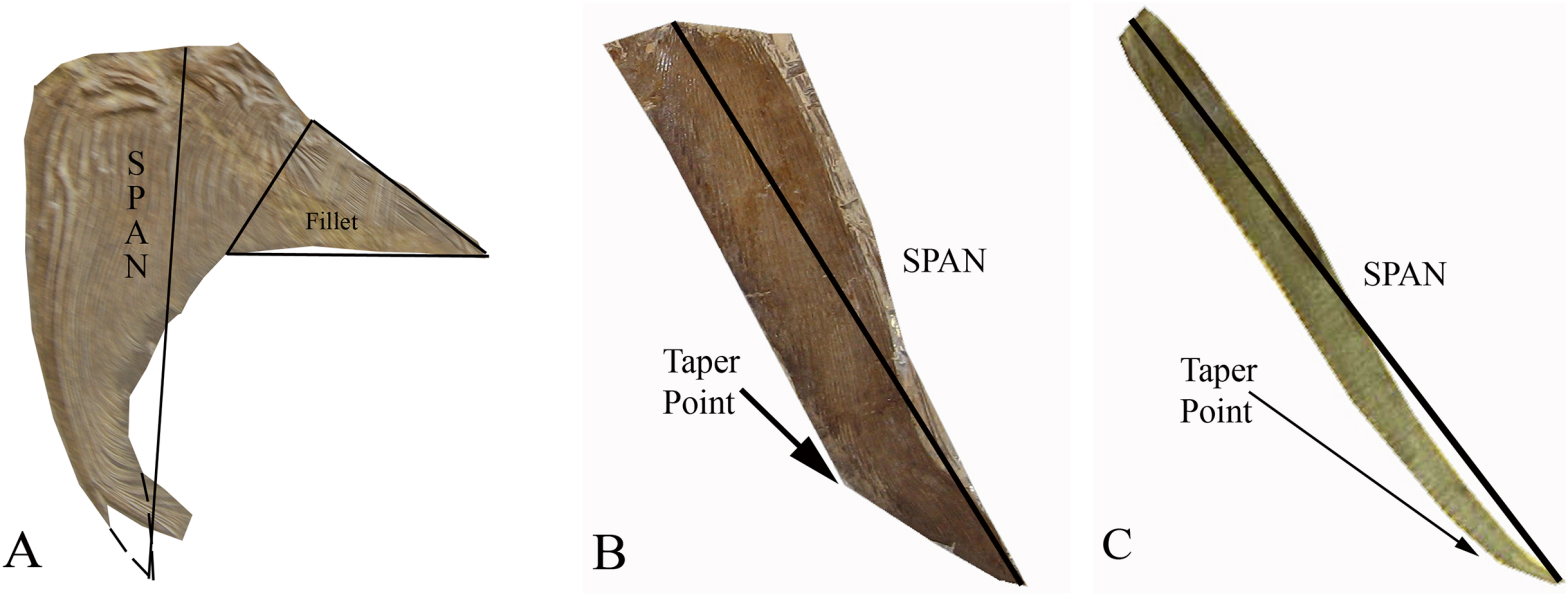
The three distinct pectoral fin morphotypes determined within the Pachycormiformes. Span line shows measurement taken for AR calculation: (A) falceform or ‘sickle’ morph (right pectoral fin of *Saurostomus esocinus* SMNS 56982) with restored position of fin-tip and fillet structure marked; (B) gladiform or ‘blade’ morph (right pectoral fin of *Bonnerichthys gladius* FHSM VP-17428) with point of transition from gradual to accelerated taper; (C) falcataform or ‘scythe’ morph (right pectoral fin of *Protosphyraena perniciosa* FHSM VP-80) with point of transition from parallel to acute taper. Figure by JJ Liston, from photographs by Soledad Gouric-Cavalli, Anthony E Maltese and Mike Everhart.

Consideration of the pectoral fins resulted in three groups of pectoral fin morphotypes (Fig. 2), with corresponding AR values. Genera tended to be morphotype specific, with few exceptions (discussed below). The morphotypes are classified according to the following descriptions (functional interpretations are derived from comparison with recognised aerodynamic equivalents, Liston & Maltese, 2016):

Type 1 Falceform or ‘Sickle’ morph (Fig. 2A): Pectoral fins that possess a distinct posterior fillet at their proximal boundary. This fillet consists of a narrow strip of lepidotrichia at the origin of the pectoral fin, projecting rearward and tapering along the junction with the body. Functionally, such a structure would reduce interference drag, increase the pectoral fin surface and consequently wetted area drag potentially as much as doubling the chord for a given span (Gough, 1928; Sherman, 1947). This lowers AR, with the sample group having a Mean (average) value of 3.88 (Mode of 4–5, Median of 3.77). The right pectoral fin of *Saurostomus esocinus* SMNS 56982 is the designated archetype for falceform fins. We interpret falceform morphology as a primitive state, representative specimens ceasing to appear in the fossil record subsequent to the Late Jurassic (Kimmeridgian-Tithonian of Solnhofen).

Type 2 Gladiform or ‘Blade’ morph (Fig. 2B): Pectoral fins lacking substantial proximal posterior expansion at the fin-body junction. Anterior and posterior margins nearly parallel at origin, tapering quite gradually but constantly to the distal tip. Reduced proximal chord results in a higher AR fin, with the sample group displaying a higher Mean (average) value of 4.35 (Mode of 3–4, Median of 4.01). The right pectoral fin of *Bonnerichthys gladius* FHSM VP-17428 is the archetype for gladiform fins. These fins are generally broad, of the type previously referred to as a tightly-bound series of parallel sticks for *Asthenocormus* as well as other as yet undescribed suspension feeding pachycormids (Liston, 2008; Liston & Friedman, 2012; Liston et al., 2012). In terms of fin extent, the gladiform fin will have a much longer span, when compared with a falceform fin of identical AR. All suspension-feeding pachycormids fall within the gladiform morphotype, with the basic planform common from the Early Jurassic until the very end of the Cretaceous.

Type 3 Falcataform or ‘Scythe’ morph (Fig. 2C): Lacking substantial proximal posterior expansion at the fin-body junction, the leading and trailing edges extend roughly parallel distally for the majority of the fin length before tapering, sometimes curving posteriorly beyond the trailing edge origin point. Though not universal (*Protosphyraena nitida* is the exception), leading edge ornamentation is common. The long slender shape results in an exceptionally high AR with a Mean (average) value in the sample group of 14.40 (Mode of 14–15, Median of 14.33), with the archetype right fin of *Protosphyraena perniciosa* FHSM VP-80 attaining nearly 15. Falcataform fins are ubiquitous on Cretaceous pachycormid pursuit predators, a lifestyle inferred not just from the large sharp dentition, but also from the similarities of the *Protosphyraena* bodyplan to contemporary istiophorids such as marlins and swordfishes (Block, Booth & Carey, 1992), with their high AR pectoral fins enabling a high degree of maneuverability, especially in tight turns, in combination with their high AR semilunate tails for high velocity.

The AR range and morphotype of each genus (see Table 1) was found to be moderately conservative and constrained, except in the cases of *Protosphyraena* and *Pachycormus*, where they were found to be unusually wide-ranging. However, the Early Jurassic *Pachycormus*, which is ubiquitous within museum collections and is easily the most common genus in the sample (see Table 2 for shape analysis breakdown), has a wide overlap in its AR range with other genera, whereas the Late Cretaceous *Protosphyraena* has no AR overlap with other genera. The range of AR for *Pachycormus* is independent of SL, therefore is interpreted as a wider ecological and possibly taxonomic disparity within the genus, than is currently recognised (Wretman, Blom & Kear, 2016).

In the Early Jurassic, three genera (*Saurostomus*, *Pachycormus* and *Haasichthys*) display the falceform morphotype, with four (*Euthynotus*, *Ohmdenia*, *Sauropsis* and *Pachycormus*) displaying the gladiform morphotype. In the Late Jurassic, only one genus (*Orthocormus*) displays the falceform morphotype, with six genera (*Sauropsis*, *Hypsocormus*, *Asthenocormus*, *Pseudoasthenocormus*, *Leedsichthys*, *Martillichthys*) displaying the gladiform morphotype. By the Cretaceous, only one genus displays the gladiform morphotype (*Bonnerichthys*) and two genera (*Australopachycormus* and *Protosphyraena*) display the falcataform morphotype.

### Geometric morphometric results

**Figure 3:**
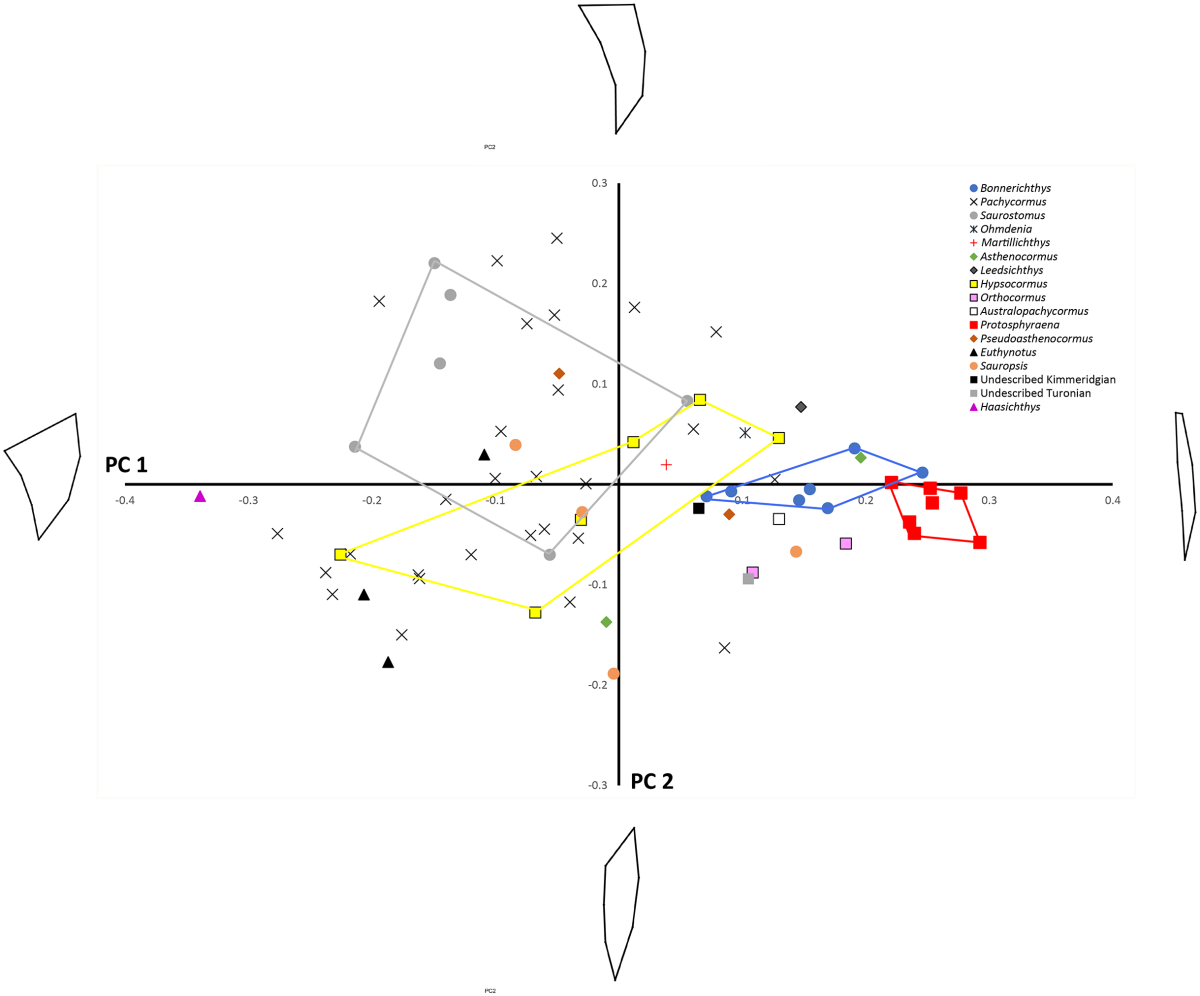
PC1 vs. PC2 plot. Principal components analysis: PC1 (*x*-axis) vs. PC2 (*y*-axis) showing distribution of sample labelled according to taxonomic affiliation. Convex hulls are erected around particular taxa of interest. Wireframe models indicate the approximate morphology at the positive and negative ends of the PC1 and PC2 axes.

**Figure 4:**
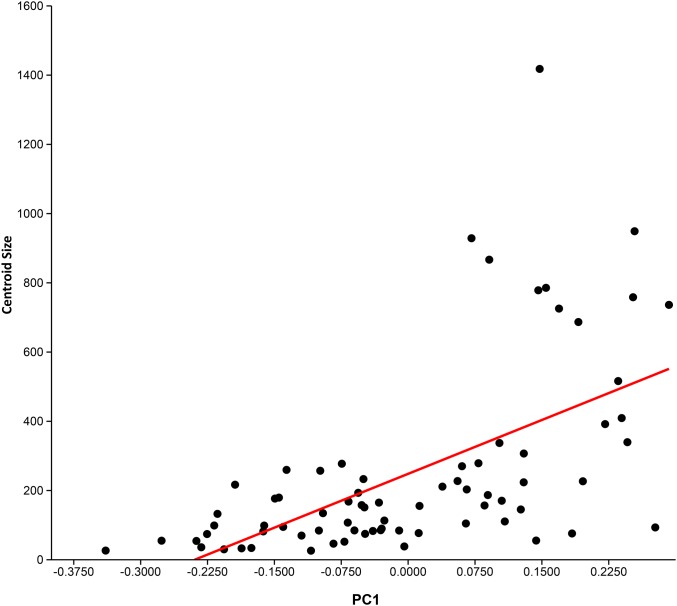
Relationship between centroid size and PC 1. PC 1 (*x*-axis) vs centroid size (*y*-axis). Ordinary least squares regression was performed on these data. A moderate-to-low, but significant (*p* = 6.9548E-08), positive relationship between centroid size and PC 1 is present, with a corrected *r*^2^ of 0.33.

Principal component analysis (see Table 3 for Eigenvalues and % variance) indicated that over 75% of the variance was accounted for by PC1 and PC2. Wireframe models on Fig. 3 indicate the approximate morphology at the positive and negative ends of the PC1 and PC2 axes. There is a low, but significant, positive relationship between centroid size and PC1 (Fig. 4), with a corrected *r*^2^ of 0.33 when least squared linear regression is performed. As such, while evolutionary allometry does partially explain PC1, the majority of shape variation on this axis is likely due to other factors, such as phylogeny or function. No other PC axis significantly correlates with centroid size.

**Table 3:** Eigenvalues and % variance for principal components analysis.

PC	Eigenvalues	% Variance	Cumulative %
1	0.02342953	54.289	54.289
2	0.00933837	21.638	75.927
3	0.00489991	11.354	87.281
4	0.00243296	5.637	92.919
5	0.00095602	2.215	95.134

Shape changes along PC1 largely relate to antero–posterior widening of the pectoral fin, especially where it attaches to the main body. On PC1 *Protosphyraena* is found at the extreme positive end and is distinct from all other genera bar a small overlap with one *Bonnerichthys* individual. *Bonnerichthys* is also found only at the positive end of PC1. *Haasichthys* is found at the extreme negative end of PC1. Shape changes along PC2 relate to how scimitar-like the fin is. Only *Pachycormus* and *Saurostomus* specimens are found at the positive end of PC2. It should be noted that the high variation seen for *Pachycormus* on PC1 vs. PC2 may, in part, be due to its relatively high sample size.

**Figure 5:**
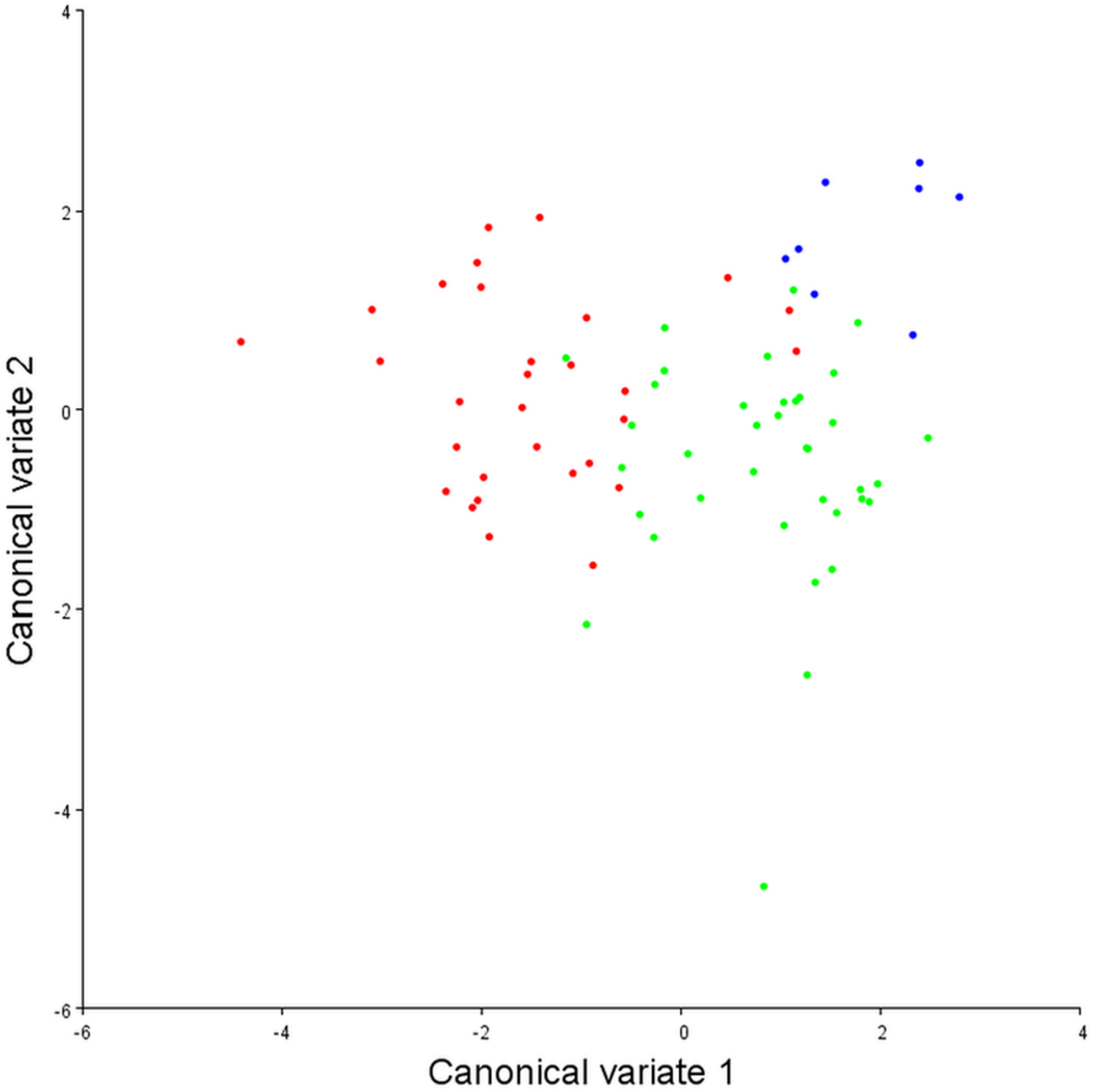
Canonical variate analysis plot. Canonical variates analysis: CV1 (*x*-axis) vs. CV2 (*y*-axis). Red dots, Morphotype 1; Green dots, Morphotype 2; Blue dots, Morphotype 3. Strong clustering is exhibited by the three pectoral fin morphotypes. On CV1 there is little overlap between morphotypes 1 and 2, and virtually no overlap between morphotypes 1 and 3.

To assess the robustness of the three pectoral fin morphotypes (see above), a canonical variate analysis (CVA) plot was conducted (Fig. 5), which showed strong clustering for the three morphotypes, with little overlap between morphotypes 1 and 2 on the CV1 axis, and virtually no overlap between 1 and 3. On axes 2 and 3 the groups overlap a little more, although CV2 mainly separates morphotypes 2 and 3.

With DFA (Table 4), morphotypes 1 and 2 have a high percentage of correct classifications. Only 13% of morphotype 1s get classed as morphotype 2s, and 11% of morphotype 2s classed as morphotype 1s. For morphotype 1 vs. morphotype 3, only 6.7% of the morphotype 1s were misclassified as morphotype 3s, and 100% of the morphotype 3s were correctly classified. When morphotype 2s and morphotype 3s are compared, only 3% of morphotype 2s are mis-classified. Morphotype 3 classifies correctly 100% of the time, although it does have a much smaller sample size. Overall, in conjunction with the CVA, the results from the DFA show that these three morphotypes are robust categories with high rates of correct classification.

**Table 4:** Cross validation percentages for discriminant function analysis.

Discriminant Function (using cross validation)—1 vs. 2	1	2
1	80% (24/30)	20% (6/30)
2	19% (7/37)	81% (30/37)
Discriminant Function (using cross validation)—1 vs. 3	1	3
1	83.3% (25/30)	16.7% (5/30)
3	0% (0/8)	100% (8/8)
Discriminant Function (using cross validation)—2 vs. 3	2	3
2	91.9% (34/37)	8.1% (3/37)
3	0% (0/8)	100% (8/8)

**Figure 6:**
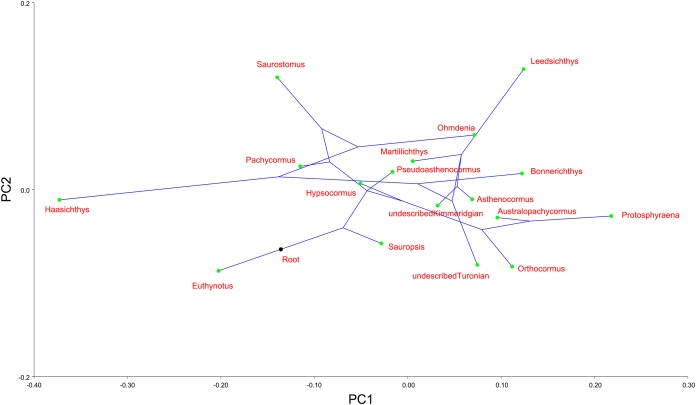
PC1 vs. PC2 for taxon means. PC1 vs. PC2 for taxon means with a composite tree mapped into the phylomorphospace to investigate shape relationships in a phylogenetic context.

Finally, we mapped a composite phylogenetic tree (the Nexus file text is included in Appendix 1), based on previous work (Schumacher et al., 2016), into the morphospace of a PCA of the genus means for the whole sample (Table 2; Fig. 6) in order to investigate shape relationships in a phylogenetic context and to adjust for any overweighting of taxa caused by the disparity in the relative numbers of specimens. There is some phylogenetic structure, particularly for the *Australopachycormus*, *Orthocormus*, *Protosphyraena* clade, which occupies a discrete part of the morphospace at the positive end of PC1 and the negative end of PC2, but there are also multiple examples of convergence. Most notably *Saurostomus* and *Leedsichthys* both converge on the positive end of PC2, echoing Woodward’s comments on the similarity between the two taxa (Woodward, 1916). *Bonnerichthys*, *Leedsichthys* and *Protosphyraena* also all converge on the positive end of PC1, and *Saurostomus*, *Haasichthys*, and *Euthynotus* do the same at the negative end of PC1.

## Discussion

**Figure 7:**
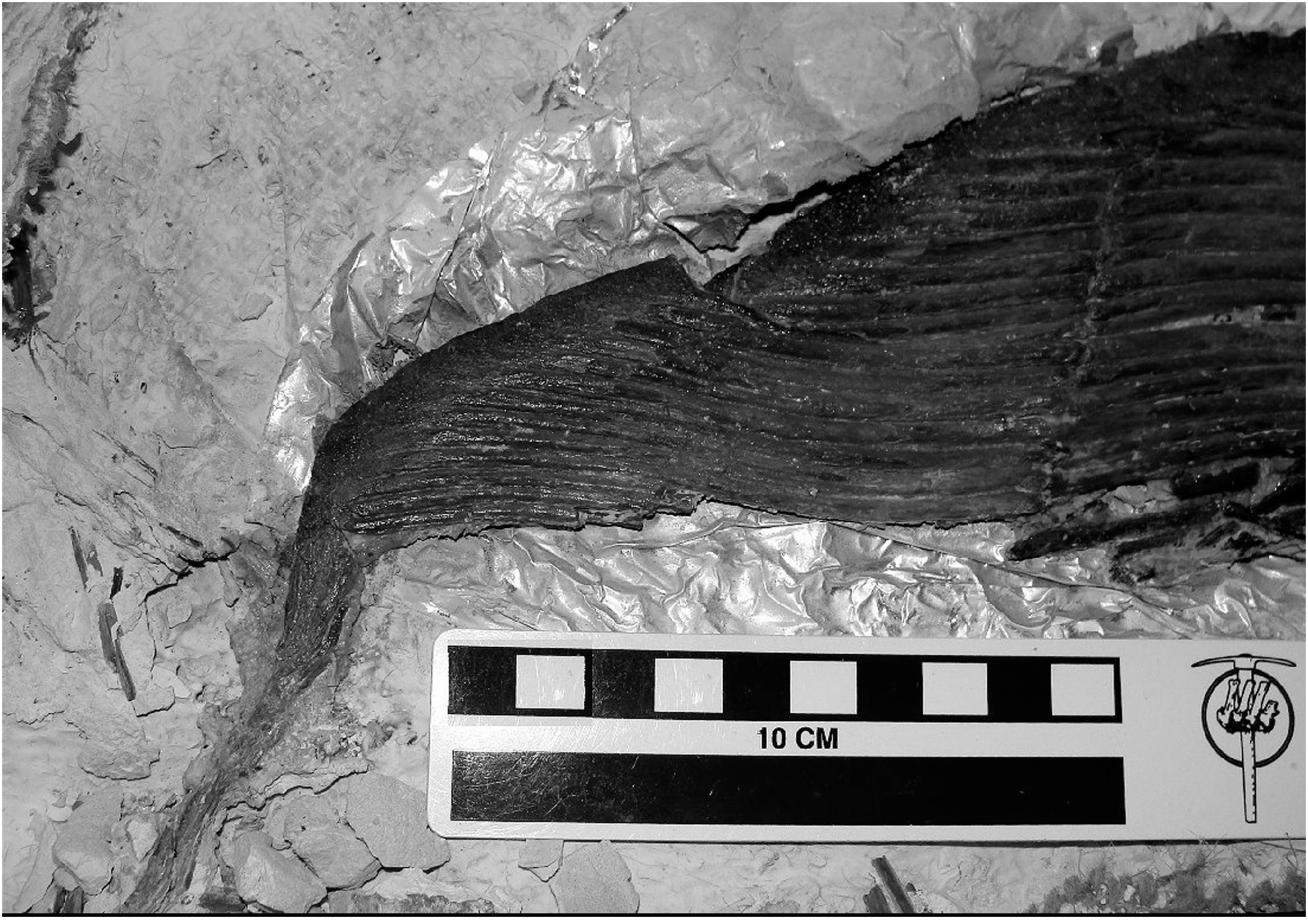
Detail of extended structure in specimen FHSM-VP 17428. Structure is theorised to function by reducing the induced drag of wingtip vortices, so that more lift generation can occur at the tips of the pectoral fins. This structure is also observable in the opposite pectoral fin of this specimen, and other *Bonnerichthys gladius* specimens e.g. RMDRC 14-017, KUVP 465, see also Friedman et al. (2010) Fig. S8. Scale bar = 100 mm. Photograph, Mike Everhart.

Arratia & Schultze (2013: p.115) noted that: ‘it is a mistake to assume that a feature observed in one pachycormiform must be present in others’ (p.115). Regardless of preservational quality, this is fundamentally true with regard to the shape of the pectoral fin. When Arthur Smith Woodward noted trends of scale size and notochord segmentation in his 1895 catalogue, he appears to have paid less attention to defining pectoral fin shape within the group, and was equivocal regarding the position of the species *esocinus* as within *Pachycormus* or outside as its own genus *Saurostomus* (Woodward, 1895). The pectoral fins of *Pachycormus* and *Saurostomus* specimens are often associated with old labels in German collections, colloquially referring to them as ‘Flugfisch’ or ‘Vogelfisch’ (‘flying fish’ or ‘bird fish’, J. Liston, 2002, personal observation, e.g. SMNS), both terms indicating their unusual and prodigious wing-like pectoral fins to palaeontological collection curators. Woodward had changed his position by the time he described specimen NHM UK PV P.11126 (Woodward, 1916), within which he was not only struck by the similarity of some of the animal’s bones to that of *Leedsichthys*, but also remarked on the pectoral fins in terms of being functional tactile organs, to an extent that indicates how remarkable this feature was within his prodigious experience. Indeed, he described one of them in terms that closely resemble the description of an ipnopid ‘bottom-walking’ fish (Woodward, 1916). That specimen of *Saurostomus* (NHMUK PV P.11126) also stands out in this survey, as having a slightly high AR, and also a subtly different fin shape, with a more attenuated leading fin ray, extending rearward beyond the rest of the fin. This also echoes another *Saurostomus* specimen SMNS 12576, which appears to share the same characteristic, but the feature was interpreted during initial assessment as a sign of preservational or preparational artefact in that specimen, so it was not included in this study. In retrospect, this was a mistake, particularly given the occurrence of a very similar morphological characteristic in another gladiform taxon, *Bonnerichthys*, which preserves a functional equivalent to the attenuated leading pectoral fin ray orientated backwards and extending beyond the margin of the trailing edge of the fin (Fig. 7) (Liston & Maltese, 2017). Arguably, this extended structure might fulfil a similar hydrodynamic role in both cases, acting like winglets (Fish, 2006) to generate ‘wash in’ and reduce the induced drag of wingtip vortices so that more lift generation occurred at the tips of the pectoral fins (Blevins & Lauder, 2012). Differences in feeding behaviours and swimming patterns are reflected in morphological differences in pectoral fins (Motta et al., 1995). As such, the gladiform morphotype is noted as the morphotype that contains all suspension feeding pachycormids. This is unsurprising, as an effective pectoral fin-based lifting surface is critical for lift generation for a slow-travelling suspension-feeder (a low feeding speed is essential in order to not create a bow-wave effect, which might act to push food away from the path of the suspension-feeding fish, thereby restricting the amount of food entering the mouth). Morphology has been remarked on as a poor predictor of diet, with the noteworthy exception of midwater ‘planktotrophic filter-feeders’, for which a common suite of characters (including pointed pectoral fin, forked caudal fin, long closely-spaced gill rakers) has been highlighted (Motta et al., 1995), all features that *Leedsichthys* and other edentulous (toothless) pachycormids possessed (Liston, 2007, 2008). It is thus similarly unsurprising that Friedman’s Toarcian sister-taxon to all edentulous Pachycormiformes, *Ohmdenia*, also is gladiform (Friedman, 2012). Although much has been speculated about the specific diets of the large suspension feeding pachycormids (Liston, 2013; Tajika, Nützel & Klug, 2018), the only genus to have received mathematical calculations of the size of food particles that might be extracted using their gill rakers is the largest (Liston et al., 2013), *Leedsichthys*. For this genus, the one to two mm calculated optimal food particle size for the gill rakers would include a large proportion of both copepods (Liston, 2007) and ammonitella/belemnitella (ammonoid and belemnite hatchling) size (0.5–1.5 mm, Tajika, Nützel & Klug, 2018). The latter proposed diet is consistent with the records of ancestral toothed pachycormids preying on cephalopods (Přikryl et al., 2012).

The pectoral fin assigned to *Australopachycormus* is not only separated chronologically and spatially from occurrences of the similar *Protosphyraena*, but the AR falls outwith the range of both *Bonnerichthys* and *Protosphyraena*, which argues for a distinction to be maintained between the genera *Australopachycormus* and *Protosphyraena*.

**Figure 8:**
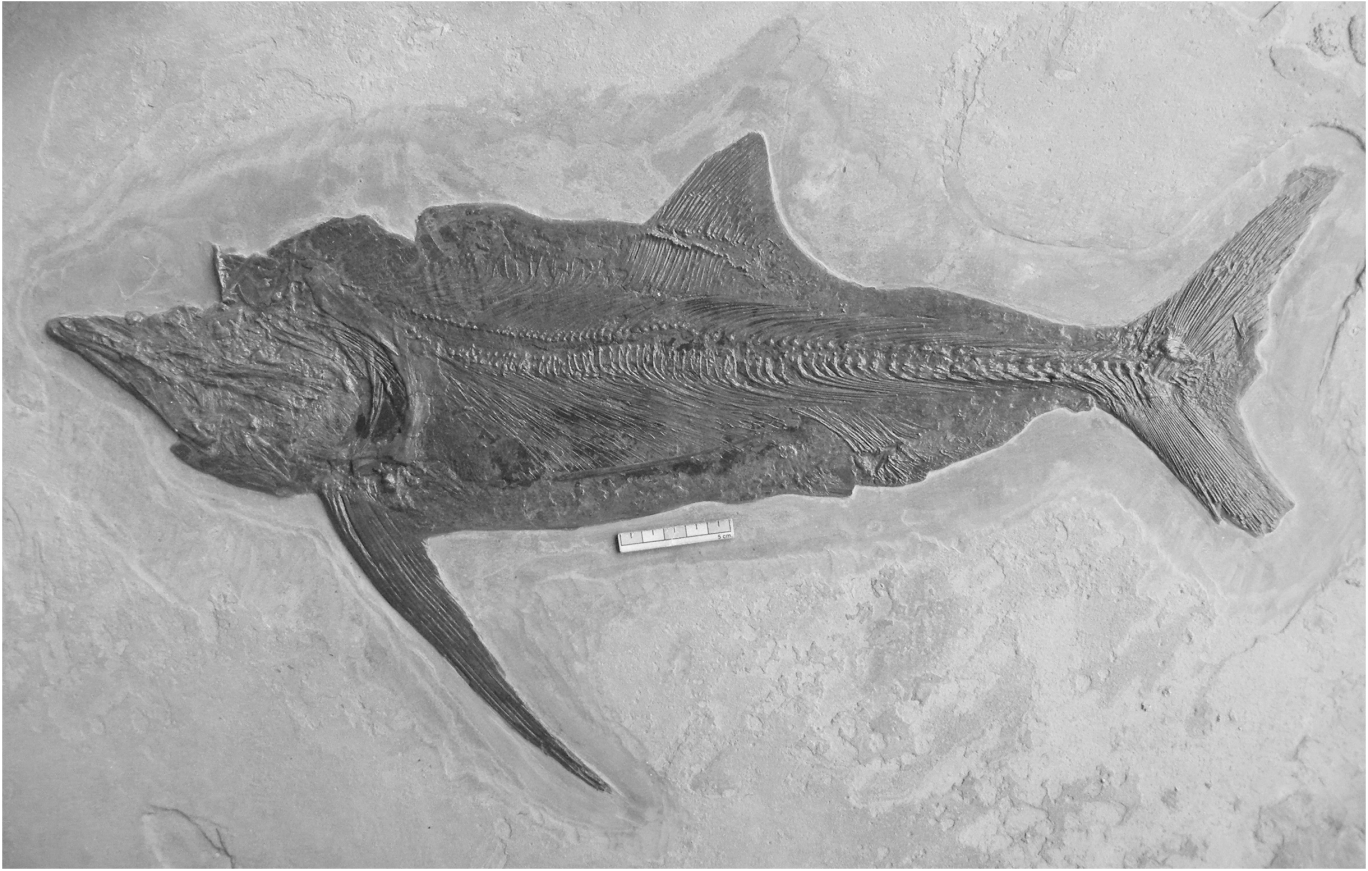
The highly unusual *Pachycormus* specimen IRSNB Vert-00-133. With unusually attenuated pectoral fins giving an extremely high aspect ratio, it is distinct from all other examples of *Pachycormus*. Scale bar = 50 mm. Photograph, Jeff J Liston.

Aspect ratio ranges were generally conservative for monospecific genera (e.g. *Euthynotus*, *Asthenocormus*), with the exceptions of *Bonnerichthys* (2.3) and *Saurostomus* (3.5). For genera with two or more species sampled, the ranges increased significantly, culminating in ranges of approximately nine in both the trispecific *Protosphyraena* and *Pachycormus*. As a high AR falcataform, this range was less surprising for *Protosphyraena* than *Pachycormus*. *Pachycormus* was recorded in the survey as a general genus rather than individual species, as any previous species level identifications associated with specimens in collections were invariably unexplained and therefore deemed unsafe. In addition, it is known that many museum collections organised by non palaeoichthyologists use the flawed taxonomy presented in ‘Das Holzmadenbuch’ (Hauff & Hauff, 1981) as a simple way to identify specimens in their collection. It was thus interesting to see the resulting spread of AR and fin types across this genus from around two thirds Type 1 to a third Type 2, and even an isolated *Pachycormus* with a falcataform grade of AR (IRSNB Vert-00-133) (Fig. 8). Interestingly, comparison of SL with AR revealed no pattern reflected in size within the genus *Pachycormus*, whereby a change in AR could have been interpreted as part of a series of ontogenetic sequences represented within the data. It is worth noting that there is no strong relationship between shape and size of the pectoral fins across the pachycormids, as demonstrated by a plot of centroid size against PC1 (Fig. 4), resulting in a corrected *r*^2^ of 0.31952, a moderate positive allometric relationship, whereas there is no significant relationship for centroid size against PC2.

This surprising result indicates a highly diverse Toarcian genus (similarly found by Cawley et al., 2019), and suggests the possibility of *Pachycormus* occupying a wide variety of lifestyles and ecological spaces. It seems that it is time for a large-scale review of everything so far identified as part of the genus *Pachycormus*, in order to assess the adequacy of current species definitions, and particular attention should be paid in that review to IRSNB Vert-00-133.

The falcataform morphotype is interpreted as one for high hydrodynamic performance, with increased manoeuvrability resulting from a biological hydrofoil equivalent to the near-elliptical planform of the humpback whale, *Megaptera novaeangliae*, as an optimal shape for uniform distribution of lift over span, and for incurring the least induced drag (Fish & Battle, 1995). Another similarity between the planform of falcataform pachycormids and the humpback whale is the common falcataform leading edge ornamentation, paralleled by the flipper tubercles of the humpback whale, which produce a scalloped appearance on their leading edges. Both features appear to have a functional effect of reducing drag on the biological hydroplane of each animal, and thus maintain lift at high angles of attack (Fish & Battle, 1995; Fish et al., 2011).

### Limitations of aspect ratios as a tool

It should be noted that the calculation of an AR for an isolated pectoral fin does not have a direct bearing on the AR of an individual specimen’s lifting surface. As noted by Liston (2007), this involves both pectoral fins, as well as the area of the ventral surface of the body of the animal between those fins, so as to give a measure of the full ‘wing’ lifting surface. Very few fossil fish preserve with SL, body width, and well-preserved pectoral fins, so this would be a far less informative measurement to attempt to take across the group. Indeed, only three specimens in our survey could be estimated in this way: a falceform *Saurostomus* (SMNS 50736, AR 5.22), a gladiform *Leedsichthys* (PETMG F174, AR 13.12) and a falcataform *Protosphyraena* (RMDRC 03-005, AR 9.87). The first two specimens lack an SL (the *Leedsichthys* specimen has been estimated at around eight m long (Liston et al., 2013), on the basis of pectoral fin size, although this has recently been reassessed to be nearer nine and a half m), and although the articulated *Protosphyraena* is unique, it has one pectoral fin missing, so measurements have to be made to the midline of the body, then doubled, in order to obtain a measurement of the full lifting surface.

Nursall (1958) dismissed the use of ARs as a phylogenetic tool, noting a pattern of swimming ability, tail-form and musculoskeletal changes varying together along different evolutionary paths, in four discrete groups defined by character combinations broadly similar to those present in the swimming modes already referred to. However, in contrast to Nursall’s broad phylogenetic objections, this is a narrow survey within a tightly defined group, looking for signs of heretofore unrecognised diversity, and possible lifestyle indicators.

### Pectoral fins, absence of swim bladders and buoyancy

As Liston (2007) noted, pachycormids appear to have lacked swim bladders (noted as strongly associated with reduced skeletal ossification in teleosts by Freedman & Noakes, 2002; Schumacher et al., 2016), so needed to expend additional energy swimming forward and compensating for drag from their large pectoral fins maintaining lift and thereby their position in the water column. This has also been argued as part of the driving force behind the trend to reduced skeletal ossification, thereby decreasing density in pachycormids of increasing SL (Liston, 2007). However, there were distinct benefits in terms of possessing a more compact and streamlined body including reduction of surface drag, dispensing with the energy intensive *rete mirabile*, and being able to change depth with comparative ease (Helfman et al., 2009). This means that, in addition to being highly manoeuverable predators, other pachycormids would find it comparatively easy to follow plankton blooms changing depth as part of their diurnal cycle.

It is another indication of a broader strategy of combatting negative buoyancy, that the pachycormids are defined by their characteristic large pectoral fins (Woodward, 1916). Although pectoral fins with such a large surface area might be interpreted as possible tools for ‘non-body’ swimming, it is clear that regardless of whether or not these fins might have been able to rotate, they could not (given their rigid, branched, unjointed and unsegmented nature) generate the ‘feathering’ or other necessary rowing motions required for this activity (Blake, 1983; Aiello et al., 2018). This is in contrast to the hinged pectoral fins of the sturgeon (Wilga & Lauder, 1999) whose function was misrepresented as a lifting surface, when the ability to alter the surface of the fin actually aided manoeuvering far more than it generated lift. Hay (1903) noted that unlike many bony fish, it seems that pachycormids could not fold their fins out of the slipstream against their body. What appears more likely when one regards the pectoral surfaces of suspension feeding pachycormids as ‘lifting wings’ (Webb, 1975) is that these large pectoral fins were required in order to generate sufficient passive lift from the fish’s forward motion for it to be able to maintain neutral buoyancy. Species with large pectoral fins and thus a greater lifting force relative to SL, should have a lower minimum swimming speed than those with small pectoral fins (Blake, 1983: p.157). In this context, it is worth noting the widespread presence of a lunate tail across pachycormids, and note that Lighthill (1969) looked more closely at the convergence on the form of the lunate tail by many groups of fish and other vertebrates, regarding it as a culmination in the enhancement of speed and propulsive efficiency (Lighthill, 1970). Lighthill suggested that this might be because this particular shape of tail readily sheds vertical vortex rings of a near circular shape, carrying a large amount of momentum and thus increasing power effectiveness (Lighthill, 1969). Again, a greater swimming efficiency produces a lower minimum swimming speed, which might also explain why something one might expect to be large and sluggish, like the suspension feeder *Leedsichthys*, still had a 4.32 AR tail, indicating a moderate cruising speed (Liston, 2007; Liston et al., 2013).

This was also noted for pectoral fins by Gero (1952) in terms of the power loading decreasing with increasing body mass for a given velocity, because the power varies with the surface area as the weight of muscle varies with the body mass. Less lift is required to counter sinking in fish that are smaller or that have an overall density closer to that of their habitat (Magnuson, 1978). Although we lack specific data on relative lift from the keel and the lower surface of many pachycormids, the full lifting surface figures above for the specimens of *Saurostomus*, *Leedsichthys* and *Protosphyraena* give moderately to extremely high ARs of 5.22, 13.12 and 9.87, respectively, thus giving a higher ratio of lift to drag over a similar fish with shorter pectoral fins (Magnuson, 1978). This is consistent with the observation that pectoral fins grow allometrically to lower fin loadings (Magnuson, 1978; similarly noted for plesiosaur paddles in O’Keefe & Carrano, 2005). Conversely, as larger animals tend to travel faster than smaller ones, and lift is a result of the square of the velocity, it is found that although lifting fins are comparatively larger on larger animals, they tend to not be as enlarged as one would expect for the size of the animal (Vogel, 1994).

There are other signs of moves towards greater swimming efficiency within this group: Arthur Smith Woodward noted the unusually high degree of segmentation present in the notochord (which is even present in some potentially younger specimens with poor preservation of centra components) compared for example with a specimen of *Caturus*, and also that they had minute rhombic scales (‘microlepidoti’ of Wagner, 1860; Heineke, 1906). Both of these features could be interpreted as enhancing movement through the water, through greater flexibility, and increasing effective tail movement for propulsion. Microlepidoti may have an additional benefit of acting much like shark denticles to reduce drag over the body surface of the fish (Oeffner & Lauder, 2012; Domel et al., 2018).

Although Hildebrand & Goslow (2001: p.426) have commented that ‘aquatic giants support their bodies effortlessly by flotation’, these fishes, many over a metre in SL, evidently employed a series of strategies in order to resolve their buoyancy problems.

## Conclusions

Reviews of characters traditionally associated with the group demonstrate that the presence of small, thin rhombic scales and the ‘scythe’-like pectoral fin are not pachycormid synapomorphies, and reduced ossification means that much of the skeleton in many taxa is simply not preserved. Analysis of pectoral fin shape in particular indicates three clear morphotypes, supported by geometric morphometric analysis. It is evident that this group requires a long overdue large-scale systematic overhaul in order to stabilise it. Thus, as our recognition of the distribution and range of both ‘classic’ and new pachycormid taxa worldwide has increased, so also has our need to go back and resolve the inadequate descriptions of the past, with the benefits of a much-expanded knowledge of variation in populations through the specimens that we know today.

## Supplemental Information

10.7717/peerj.7675/supp-1 Supplemental Information 1 Nexus file text used in Geometric Morphometric analyses.

10.7717/peerj.7675/supp-2 Supplemental Information 2 The location and accession numbers for each specimen.

## Institutional abbreviations

Material was reviewed from the following institutions:

AMNH: American Museum of Natural History, New York, New York, USA
BaJ: Staatliches Museum für Mineralogie und Geologie zu Dresden, Germany
BSPG: Bayerische Staatssammlung für Paläontologie und historische Geologie, München, Germany
CM: Carnegie Museum of Natural History, Pittsburgh, Pennsylvania, USA
FHSM: Sternberg Museum of Natural History (Fort Hays State Museum), Hays, Kansas, USA
Ge: Moravské zemské muzeum Brno, Czech Republic
GLAHM V: Hunterian Museum University of Glasgow, Scotland
G/PA: Utrecht University Museum, The Netherlands
GPIT: Institut für Geowissenschaften, Eberhard Karls Universität Tübingen, Germany
IRSNB: Royal Belgian Institute of Natural Sciences (Museum of Natural Sciences), Brussels, Belgium
JM-E SoS: Jura Museum Eichstätt, Germany
K: The Etches Collection Museum of Jurassic Marine Life, Kimmeridge, England
KUVP: University of Kansas, Natural History Museum, Lawrence, Kansas, USA
L: Bochum Tierpark (Leich Collection), Germany
LACM: Natural History Museum of Los Angeles County, Los Angeles, California, USA
MBF: Museum für Naturkunde—Leibniz-Institut für Evolutions- und Biodiversitätsforschung an der Humboldt-Universität zu Berlin, Germany
MNHNP (STC): Muséum National d’Histoire Naturelle, Paris, France
NHMUKPV P: Natural History Museum (London), England
NMS: National Museums Scotland, Edinburgh, Scotland
PETMG: Peterborough City Museum, England
PMU: Uppsala University, Sweden
RMDRC: Rocky Mountain Dinosaur Resource Center, Woodland Park, Colorado, USA
SenkM: Naturmuseum Senckenberg, Frankfurt, Germany
SMNK-PAL: Staatliches Museum für Naturkunde Karlsruhe, Germany
SMNS: Staatliches Museum für Naturkunde Stuttgart, Germany
T: Teylers Museum, Haarlem, The Netherlands
Tu: Musée national d’Histoire naturelle de Luxembourg, Luxembourg
UANL-FCT: Universidad Autónoma Nuevo León, Facultad de Ciencias de la Tierra, Linares, Mexico
UNSM: University of Nebraska State Museum, Lincoln, Nebraska, USA.

## Additional Information and Declarations

### Data Availability

The following information was supplied regarding data availability: The raw data is included in Table 1. The accession numbers and specimen locations are available in the Supplemental File.
