# Supplementary material for: Scythes, sickles and other blades: defining the diversity of pectoral fin morphotypes in Pachycormiformes
Source: PeerJ. 2019 Nov 7;7:e7675. doi: 10.7717/peerj.7675 (PMC6842561; doi:10.7717/peerj.7675)
Supplement: Supplemental Information 1 [file peerj-07-7675-s001.docx]

Appendix 1: Nexus file text used in GM analyses

#NEXUS

BEGIN TAXA;

TAXLABELS Euthynotus Sauropsis Pseudoasthenocormus Pachycormus Saurostomus Ohmdenia Haasichthys Bonnerichthys undescribedTuronian Martillichthys Asthenocormus undescribedKimmeridgian Leedsichthys Hypsocormus Orthocormus Australopachycormus Protosphyraena;

END;

BEGIN TREES;

TREE tree1 = (Euthynotus,(Sauropsis,(Pseudoasthenocormus,(Pachycormus,(Saurostomus,(Ohmdenia,(Haasichthys,(Bonnerichthys,(undescribedTuronian,(Martillichthys,(Asthenocormus,undescribedKimmeridgian),Leedsichthys)))))))(Hypsocormus,(Orthocormus,(Australopachycormus,Protosphyraena)))));

END;
