# Supplementary material for: Scythes, sickles and other blades: defining the diversity of pectoral fin morphotypes in Pachycormiformes
Source: PeerJ. 2019 Nov 7;7:e7675. doi: 10.7717/peerj.7675 (PMC6842561; doi:10.7717/peerj.7675)
Supplement: Supplemental Information 2 [file peerj-07-7675-s002.docx]

**RAW DATA DECLARATION**

In this table, the specimen accession number abbreviations correspond to the following 29 institutions, where they are located:

AMNH=American Museum of Natural History, New York, New York, USA; BaJ=Staatliches Museum für Mineralogie und Geologie zu Dresden, Germany; BSPG=Bayerische Staatssammlung für Paläontologie und historische Geologie, München, Germany; CM=Carnegie Museum of Natural History, Pittsburgh, Pennsylvania, USA; FHSM = Sternberg Museum of Natural History (Fort Hays State Museum), Hays, Kansas, USA; Ge=Moravské zemské muzeum Brno, Czech Republic; GLAHM V=Hunterian Museum University of Glasgow, Scotland; G/PA=Utrecht University Museum, The Netherlands; GPIT =Institut für Geowissenschaften, Eberhard Karls Universität Tübingen, Germany; IRSNB= Royal Belgian Institute of Natural Sciences (Museum of Natural Sciences), Brussels, Belgium; JM-E SoS=Jura Museum Eichstätt, Germany; K=The Etches Collection Museum of Jurassic Marine Life, Kimmeridge, England; KUVP=University of Kansas, Natural History Museum, Lawrence, Kansas, USA; L=Bochum Tierpark (Leich Collection), Germany; LACM=Natural History Museum of Los Angeles County, Los Angeles, California, USA; MBF=Museum für Naturkunde – Leibniz-Institut für Evolutions- und Biodiversitätsforschung an der Humboldt-Universität zu Berlin, Germany; MNHNP (STC)=Muséum National d'Histoire Naturelle, Paris, France; NHMUK PV P.=Natural History Museum (London), England; NMS=National Museums Scotland, Edinburgh, Scotland; PETMG=Peterborough City Museum, England; PMU=Uppsala University, Sweden; RMDRC=Rocky Mountain Dinosaur Resource Center, Woodland Park, Colorado, USA; SenkM=Naturmuseum Senckenberg, Frankfurt, Germany; SMNK-PAL= Staatliches Museum für Naturkunde Karlsruhe, Germany; SMNS= Staatliches Museum für Naturkunde Stuttgart, Germany; T=Teylers Museum, Haarlem, The Netherlands, Tu= Musée national d’Histoire naturelle de Luxembourg, Luxembourg; UANL-FCT= Universidad Autónoma Nuevo León, Facultad de Ciencias de la Tierra, Linares, Mexico; UNSM=University of Nebraska State Museum, Lincoln, Nebraska, USA.

| **Genus** | **Specimen/Collection Number** | **A/R** | **Fin Type** |
| --- | --- | --- | --- |
| *Haasichthys* | Tu228 (holotype) | 3,06 | 1 |
| *Orthocormus* | T.14836 (holotype) | 4,17 | 1 |
| *Orthocormus* | JM-E SoS Scha 2418 | 4,66 | 1 |
| *Orthocormus* | SenkM 1863 (holotype) | 1,53 | 1 |
| *Orthocormus* | BSPG.1993.XVIII-VFKO.B16 (holotype) | 2,67 | 1 |
| *Saurostomus* | NHMUK OR3731 L | 5,6 | 1 |
| *Saurostomus* | SMNS 51144 | 2,96 | 1 |
| *Saurostomus* | SMNS 56982 | 3,48 | 1 |
| *Saurostomus* | SMNS 50736 R | 2,04 | 1 |
| *Saurostomus* | SMNS 50736 L | 2,86 | 1 |
| *Saurostomus* | NHMUK PV P.11126 | 4,52 | 2 |
| *Pachycormus* | SMNS 51041 | 2,1 | 1 |
| *Pachycormus* | SMNS 55300 | 4,02 | 1 |
| *Pachycormus* | SMNS 51905 L/R | 6.28/3.14 | 1 |
| *Pachycormus* | SMNS 51199 L | 4,46 | 1 |
| *Pachycormus* | SMNS 51199 R | 4,1 | 1 |
| *Pachycormus* | SMNS 56230 | 2,94 | 1 |
| *Pachycormus* | SMNK - PAL.6680 | 3,77 | 1 |
| *Pachycormus* | PA-49.1911 | 3,03 | 1 |
| *Pachycormus* | SMNS 54835 | 3,59 | 1 |
| *Pachycormus* | NHMUK PV P.12913 | 1,34 | 1 |
| *Pachycormus* | PMU24798 | 4,68 | 1 |
| *Pachycormus* | GLAHM V7274 | 4,38 | 1 |
| *Pachycormus* | PMU24796 | 4,92 | 1 |
| *Pachycormus* | SMNS 55857 R | 4,66 | 1 |
| *Pachycormus* | SMNS 55857 L | 5,3 | 1 |
| *Pachycormus* | SMNS 6696 L/R | 6.35/4.24 | 1 |
| *Pachycormus* | SMNS 87762 R | 4,22 | 1 |
| *Pachycormus* | SMNS 15815 L | 2,33 | 1 |
| *Pachycormus* | SMNS 15815 R | 2,03 | 1 |
| *Pachycormus* | IRSNB Vert-00-133 | 10,05 | 1 |
| *Pachycormus* | NHMUK PV P.51667 | 3,54 | 1 |
| *Pachycormus* | SMNS 54835 | 3,58 | 1 |
| *Pachycormus* | NHMUK OR 20657 | 4,01 | 1 |
| *Pachycormus* | NHMUK PV P.7569 | 3,97 | 1 |
| *Pachycormus* | GPIT/OS/777 | 3,97 | 2 |
| *Pachycormus* | SMNS 58389 | 6,36 | 2 |
| *Pachycormus* | G.338-1894 | 3,06 | 2 |
| *Pachycormus* | SMNS 95430 | 3,33 | 2 |
| *Pachycormus* | Ge 30 177 | 3,86 | 2 |
| *Pachycormus* | MBF 12215 | 2,4 | 2 |
| *Pachycormus* | SMNS 95835 R | 3,43 | 2 |
| *Pachycormus* | SMNS 95835 L | 3,48 | 2 |
| *Sauropsis* | BSP.AS.VII.1089 | 6,44 | 2 |
| *Sauropsis* | NHMUK PV P.13007 (holotype) | 3,89 | 2 |
| *Sauropsis* | CM 4772 | 4,69 | 2 |
| *Sauropsis* | Tu147 | 2,32 | 2 |
| *Euthynotus* | MNHNP 821 | 3,16 | 2 |
| *Euthynotus* | MNHNP 10537 | 3,31 | 2 |
| *Euthynotus* | MNHNP 10538 | 3,97 | 2 |
| *Euthynotus* | AMNH 7540 | 3,82 | 2 |
| *Ohmdenia* | GPIT 1017/1 (holotype) | 3,84 | 2 |
| *Leedsichthys* | PETMG F.174 | 4,2 | 2 |
| *Martillichthys* | NHMUK PV P.61563 (holotype) | 5,9 | 2 |
| *Hypsocormus* | NHMUK PV P.6011 L | 3,88 | 2 |
| *Hypsocormus* | NHMUK PV P.6011 R | 4,03 | 2 |
| *Hypsocormus* | NMS 1892.55.2 | 5,42 | 2 |
| *Hypsocormus* | BSPG.1964.XXIII.524 Schernfeld | 2,21 | 2 |
| *Hypsocormus* | NHMUK PV P.6942 R | 4,1 | 2 |
| *Hypsocormus* | JM-E SoS 539 | 2,83 | 2 |
| *Hypsocormus* | JM-E SoS 3916 | 4,38 | 2 |
| *Asthenocormus* | Baj2344 | 2,96 | 2 |
| *Asthenocormus* | JM-E SoS 542 (neotype) | 3,84 | 2 |
| *Pseudoasthenocormus* | CM 5399 | 3,86 | 2 |
| *Pseudoasthenocormus* | BSP.1956.I.361 | 4,1 | 2 |
| asthenocormid (TUR) | UANL-FCT 0087 | 4,74 | 2 |
| Kimmeridgian juvenile | K.1556 | 3,99 | 2 |
| *Bonnerichthys* | UNSM 88507 | 4,58 | 2 |
| *Bonnerichthys* | FHSM VP-212 | 5,51 | 2 |
| *Bonnerichthys* | FHSM VP-17428 fin 1 | 6,87 | 2 |
| *Bonnerichthys* | FHSM VP-17428 fin 2 | 6,33 | 2 |
| *Bonnerichthys* | KUVP 465 R | 5,24 | 2 |
| *Bonnerichthys* | KUVP 465 L | 5,49 | 2 |
| *Bonnerichthys* | KUVP 60692 | 6,19 | 2 |
| *Bonnerichthys* | RMDRC 14-017 | 6,87 | 2 |
| *Protosphyraena* | FHSM VP-80 R | 14.83 | 3 |
| *Protosphyraena* | AMNH FF 21651 L/R | 16.10/14.33 | 3 |
| *Protosphyraena* | RMDRC 11-025 | 13,12 | 3 |
| *Protosphyraena* | RMDRC 14-005 | 20,05 | 3 |
| *Protosphyraena* | RMDRC 15-020 | 10,11 | 3 |
| *Protosphyraena* | RMDRC 03-007 | 13,72 | 3 |
| *Protosphyraena* | RMDRC 03-005 | 11,26 | 3 |
| *Protosphyraena* | RMDRC 03-006 | 14,71 | 3 |
| *Australopachycormus* | NHMUK PV P.73611 | 8,07 | 3 |
